# A Critical Review of Cranial Electrotherapy Stimulation for Neuromodulation in Clinical and Non-clinical Samples

**DOI:** 10.3389/fnhum.2021.625321

**Published:** 2021-02-01

**Authors:** Tad T. Brunyé, Joseph E. Patterson, Thomas Wooten, Erika K. Hussey

**Affiliations:** ^1^U. S. Army Combat Capabilities Development Command Soldier Center, Cognitive Science Team, Natick, MA, United States; ^2^Center for Applied Brain and Cognitive Sciences, Tufts University, Medford, MA, United States; ^3^Department of Psychology, Tufts University, Medford, MA, United States

**Keywords:** non-invasive brain stimulation, neuromodulation, psychiatry, human performance, electrotherapy

## Abstract

Cranial electrotherapy stimulation (CES) is a neuromodulation tool used for treating several clinical disorders, including insomnia, anxiety, and depression. More recently, a limited number of studies have examined CES for altering affect, physiology, and behavior in healthy, non-clinical samples. The physiological, neurochemical, and metabolic mechanisms underlying CES effects are currently unknown. Computational modeling suggests that electrical current administered with CES at the earlobes can reach cortical and subcortical regions at very low intensities associated with subthreshold neuromodulatory effects, and studies using electroencephalography (EEG) and functional magnetic resonance imaging (fMRI) show some effects on alpha band EEG activity, and modulation of the default mode network during CES administration. One theory suggests that CES modulates brain stem (e.g., medulla), limbic (e.g., thalamus, amygdala), and cortical (e.g., prefrontal cortex) regions and increases relative parasympathetic to sympathetic drive in the autonomic nervous system. There is no direct evidence supporting this theory, but one of its assumptions is that CES may induce its effects by stimulating afferent projections of the vagus nerve, which provides parasympathetic signals to the cardiorespiratory and digestive systems. In our critical review of studies using CES in clinical and non-clinical populations, we found severe methodological concerns, including potential conflicts of interest, risk of methodological and analytic biases, issues with sham credibility, lack of blinding, and a severe heterogeneity of CES parameters selected and employed across scientists, laboratories, institutions, and studies. These limitations make it difficult to derive consistent or compelling insights from the extant literature, tempering enthusiasm for CES and its potential to alter nervous system activity or behavior in meaningful or reliable ways. The lack of compelling evidence also motivates well-designed and relatively high-powered experiments to assess how CES might modulate the physiological, affective, and cognitive responses to stress. Establishing reliable empirical links between CES administration and human performance is critical for supporting its prospective use during occupational training, operations, or recovery, ensuring reliability and robustness of effects, characterizing if, when, and in whom such effects might arise, and ensuring that any benefits of CES outweigh the risks of adverse events.

## Introduction

Cranial electrotherapy stimulation (CES) involves delivering low-intensity (50 μA to 4 mA) electrical current via a pair of electrodes attached to bilateral anatomical positions around the head (e.g., eyelids, earlobes, mastoids, temples), with the intent of acutely modulating central and/or peripheral nervous system activity. This review describes past and present research and development efforts with this neuromodulatory technique, with emphasis on its potential for enhancing well-being in clinical contexts and optimizing or enhancing human performance in healthy, neurotypical populations.

In clinical populations, CES has been used as an adjunctive treatment for several clinical disorders including insomnia, depression, post-traumatic stress disorder, and anxiety (Gunther and Phillips, 2010; Bracciano et al., 2012; Morriss and Price, 2020). While the precise mechanisms underlying putative CES effects on clinical disorders remain elusive, proposed effects include modulation of central and peripheral nervous systems, altering resting state and limbic system activity, increasing cortical alpha-band activity, and modulating the release of neurotransmitters and downstream hormones including catecholamines and glucocorticoids (Schroeder and Barr, 2001; Feusner et al., 2012; Qiao et al., 2015; Wagenseil et al., 2018; Yennurajalingam et al., 2018; Wu et al., 2020).

In healthy populations engaged in high-stakes occupational contexts and tasks, performance enhancement can occur during learning or training, and prior to, during, and/or after occupational task performance. Indeed, neuromodulation approaches hold potential to accelerate learning and training, but also acutely modulate task performance and assist in rest, recovery, and reset phases. For example, an emergency first responder might incorporate neuromodulatory techniques to accelerate the learning of new procedural skills, modulate stress responses during high-stakes operations, or to assist emotion regulatory processes following exposure to stress. In these scenarios, CES may carry potential to help sustain or improve behavioral outcomes related to occupational performance including vigilance, perceptuomotor control, situation awareness, and emotion regulation.

To examine whether CES may carry potential to reliably alter brain activity, physiology, neurotransmitters and hormones, or behavior in clinical and non-clinical populations, we critically review extant scientific outcomes from studies examining CES, detail mechanistic models of CES effects, and reveal several methodological strengths and weaknesses of the existing literature. The report concludes with paths forward for CES research and potential application to occupational performance in healthy, neurotypical populations.

## A Brief History of CES

The first CES device, the Somniatron, was developed in the Soviet Union in the early 1900s and delivered 1–4 mA alternating current at 100 Hz via two electrodes attached to the eyelids (Robinovitch, 1914). The Somniatron was used to induce analgesia and sleep in patients with insomnia. In 1973, the first CES device was marketed in the United States without formal regulatory oversight, the Electrosone 50, for inducing relaxation and sleep (Kirsch et al., 2014). The Electrosone delivered alternating current at variable pulse frequency (up to 4,000 Hz) with 2 mA to 8 mA intensity; the device was portable and battery-operated, and electrodes were placed on the eyelids and mastoids.

Three years after the release of the Electrosone, the United States Food and Drug Administration (FDA) began regulating medical devices. In 1978, the Neurotone 101 became the first FDA-approved CES device, delivering up to 1.5 mA intensity at 50–100 Hz (Guleyupoglu et al., 2013) via electrodes placed on the supraorbital ridge and mastoid. The device was marketed for the treatment of anxiety, depression, and insomnia. In the years that followed, several CES devices were developed and marketed in the United States, including the Pain Suppressor, Transcranial Electrostimulator, Electrodorm, Fisher Wallace Cranial Electrical Stimulator, and the Alpha-Stim (Shekelle et al., 2018a).

The United States Food and Drug Administration (FDA) classifies medical devices into three Classes: Class I, II, and III, each with their own regulatory controls (Peña et al., 2007). The level of regulatory control increases across the three Classes, with Class I requiring general controls, Class II requiring special controls, and Class III requiring premarket approval. Class I products are generally low risk and not intended for supporting or sustaining life or preventing health-related impairment; examples include bandages, electronic toothbrushes, and stethoscopes. Class II products are generally moderate risk and have sustained contact with a patient, and general controls are not sufficient for ensuring device safety or efficacy; examples include syringes, contact lenses, and absorbable sutures. Class III products are generally high-risk and have unproven safety or efficacy, and have sustained and potentially life-supporting contact with a patient; examples include pacemakers, defibrillators, and medical implants. From the 1980's through early 2000's, many of the original CES devices were regulated as Class III devices by the United States Food and Drug Administration (FDA).

More recently, the FDA issued a final order (Docket No. FDA-2014-N-1209) to classify CES devices marketed to treat anxiety or insomnia as Class II (special controls) medical devices. A Class II designation is given by the FDA for devices for which general controls are insufficient to provide reasonable assurance of the safety and effectiveness of the device (21CFR860.3). In contrast, CES devices marketed to treat depression are classified as Class III medical devices, requiring additional regulatory oversight due to potential unreasonable risk of illness or injury (21CFR860.3). As part of the FDA regulation of CES devices, only licensed medical practitioners can order patient use of a CES device.

Despite this regulation, about a dozen devices are currently available for consumer purchase in the United States, varying in price from ~$300–5000USD. Technically, all available devices can only be ordered by licensed medical practitioners, or by patients who complete a low-cost telemedicine visit. Note that our research group has been able to readily procure CES devices for research purposes, specifically the Alpha-Stim M, by stating our research purpose and without demonstrating licensure. Below are some examples of currently marketed CES devices:

1. Alpha-Stim M (Electromedical Products International, Inc.)
  a. Output: Bipolar asymmetric rectangular wave at 0.5, 1.5, and 100 Hz, up to 600 μA.
  b. Electrodes: Ear clips.
  c. URL: https://www.alpha-stim.com/product/alpha-stim-m-microcurrent-cranial-electrotherapy-stimulator/.

2. Alpha-Stim 100 (Electromedical Products International, Inc.)
  a. Output: Bipolar asymmetric rectangular wave at 0.5, 1.5, and 100 Hz, up to 600 μA.
  b. Electrodes: Ear clips.
  c. URL: http://alphachoices.com/as100.html.

3. Fisher Wallace Stimulator (Fisher Wallace Laboratories, Inc.)
  a. Output: Symmetrical biphasic square wave at 15, 500, and 15k Hz, up to 4 mA.
  b. Electrodes: Sponge electrodes, mounted on temples.
  c. URL: https://www.fisherwallace.com/.

4. CES Ultra (Neuro-Fitness, LLC)
  a. Output: Modified square wave at 100 Hz, up to 1.5 mA.
  b. Electrodes: Woven electrodes with conductive gel, mounted on temples.
  c. URL: https://www.cesultra.com/.

5. Caputron MindGear (Caputron Medical Products, LLC)
  a. Output: Symmetrical biphasic square wave at 0.5 Hz, up to 1.5 mA.
  b. Electrodes: Ear clips or self-adhesive electrodes for temples.
  c. URL: https://caputron.com/collections/mindgear.

6. Neurocare NeuroMICRO (Neurocare, Inc.)
  a. Output: Modified square DC biphasic pulses at 0.3, 8, and 80 Hz, at 12V.
  b. Electrodes: Carbon electrodes with conductive gel, mounted on temples.
  c. URL: http://neurocare.com/neuromicro-cranial-electrotherapy-stimulation/.

## Clinical Applications of CES

CES has predominantly been used for the relief of symptoms accompanying three clinical disorders: insomnia, anxiety, and depression. While hundreds of studies have been published examining the effects of CES on insomnia, depression, and anxiety, most are inadequately designed and show a high risk of bias according to the Cochrane criteria (Higgins et al., 2011). To mitigate these biases, in our review we primarily consider placebo-controlled randomized clinical trials with objective measures, similarly to what was done in recent reviews by the Department of Veterans Affairs (Shekelle et al., 2018a,b).

### CES for Clinical Insomnia

The four sham-controlled randomized clinical trials using CES to treat insomnia reveal inconsistent results. One study involving 57 active-duty service member participants showed no significant change in hours of sleep time following a 5-day (60-min/day) CES treatment (100 μA, 0.5 Hz) relative to sham (Lande and Gragnani, 2013). This study used the Alpha-Stim SCS device with two electrodes attached to bilateral earlobes, and the sham condition involved an inactive device. No comparative (active vs. sham) assessment of cutaneous perception was reported. The authors suggested the CES treatment may not have been sufficient intensity (i.e., in microamps or duration) to induce reliable effects on sleep, though to our knowledge no follow-up study was conducted.

A second study involving 10 participants showed significant reduction of sleep latencies measured with electroencephalography (EEG) (Weiss, 1973). In this study, CES treatment involved a 24-day (15-min/day) CES treatment (500 μA, 100 Hz), delivered using the Electrodorm 1 device with an array of electrodes placed above the eyes and on the nape of the neck. The sham condition involved an initial stimulation and habituation phase, and then disabling the device. A report of cutaneous sensation was gathered to ensure that each participant felt a “tingling” sensation during the habituation phase, and post-treatment reports of persistent sensations (tingling, prickling, burning) were similar between the active and sham groups. Another study involving 19 psychiatric patients showed significant reduction of global insomnia ratings with a 14-day CES treatment vs. sham, using the Electrosone-50 device with electrodes placed over the orbital and mastoid areas (30-min/day; 100–250 μA, 50–100 Hz) (Feighner et al., 1973). Like the procedure used by Weiss (1973), the sham condition involved an initial stimulation phase followed by disabling the device; no comparative assessment (active vs. sham) of vestibular or cutaneous sensation was reported. Note that insomnia ratings only improved at the 15-day timepoint (not at days 1 or 26, and also not at 1-month follow-up).

A fourth double-blind randomized placebo-controlled study involving 40 females without sleep disorders showed no significant effect of CES (*n* = 25) vs. sham (*n* = 15) using the Alpha-Stim 100 device with bilateral earlobe electrodes (60-min; 100 μA, 0.5 Hz) on any measures of sleep latency or quality (Wagenseil et al., 2018). In this study, the sham condition involved an inactive device, and no measures of vestibular or cutaneous sensation were reported. While there were no significant effects of CES on sleep latency or quality, there was some limited evidence for an effect in the EEG α band as measured using polysomnography. Specifically, there was a significant decline of low-frequency (8–10 Hz) α band peak frequency, though this pattern only arose at two EEG electrode sites and was not found in the high-frequency (10–11 Hz) α band. There was no alteration of α band power across any frequency range, and the authors suggested that the EEG peak frequency results warranted replication given their apparent specificity.

### CES for Clinical Depression

A review of CES applications for depression revealed a severe lack of rigorous randomized placebo-controlled clinical trials, and a tendency to use non-standard instruments for diagnosing and monitoring depression symptoms (Kavirajan et al., 2014). In fact, of the 270 published reports on CES effects on depression, none of them reached the Cochrane quality criteria (Higgins et al., 2011). This was largely due to a failure to use standardized diagnostic criteria (e.g., the Beck Depression Inventory; BDI), lack of sufficient participant blinding (e.g., no cutaneous perception in sham group), high rates of comorbidity (e.g., fibromyalgia, anxiety, dementia, substance abuse, head injury), or consideration of only institutionalized patients with severe refractory depression (Kavirajan et al., 2014).

Two relatively high-quality publications are worth mentioning. In the first study, a group of 16 participants with depression received either sham or active CES using the Fisher Wallace Cranial Stimulator device with two electrodes placed over bilateral temples (2 mA, 5–15,000 Hz) for 10 days (20-min/day), in a randomized, double-blind, placebo-controlled design (McClure et al., 2015). The sham group received active stimulation until the participant reported a tingling sensation on the scalp, and then the device was disabled for the remainder of the session; no details were reported regarding potential differences in vestibular or cutaneous sensations between the control and sham groups. Results showed significant reductions in depressive symptoms for the active but not sham CES group at the end of week 2, measured using the BDI. These results should be interpreted with caution, however, given the relatively small sample size (active *n* = 7, sham *n* = 9).

In the second study with a larger sample size, a group of 30 participants with depression received either sham or active CES using the Fisher Wallace Cranial Stimulator (FW-100; 1–4 mA, 15–15,000 Hz) for 15 days (20-min/day), in a randomized, double-blind, placebo-controlled design (Mischoulon et al., 2015). According to the authors, this device was used with two bilateral electrodes placed over bilateral regions of the dorsolateral prefrontal cortex (dlPFC); however, as seen in Figure 2 of their report, it appears the electrodes were placed inferior to the bilateral temples. This is an important distinction because a CES device with electrodes places over the dlPFC would more accurately constitute transcranial alternating current stimulation (tACS) rather than CES. Sham devices were inactive, and there was no reporting of possible vestibular or cutaneous sensation differences between conditions. Results showed no significant differences in depressive symptoms for the active vs. sham CES groups at the end of week 3, measured using the Hamilton Rating Scale for depression (HAM-D-17).

### CES for Clinical Anxiety

The effects of CES on anxiety are marginally more reliable than effects on depression. To date, five randomized, double-blind, placebo-controlled studies have been conducted, generally showing support for CES in reducing symptoms of anxiety (Shekelle et al., 2018a). These studies suffer the limitation of clinical disorder comorbidity, with most participants diagnosed with not only anxiety (i.e., generalized anxiety disorder) but also depression, post-traumatic stress disorder, and/or insomnia. Additional limitations include the use of antiquated devices (e.g., Neurotone 101 or Electrosone-50) that are no longer available for investigation, inconsistent electrode placement (head, ears, face), lack of standardized instruments for measuring anxiety, and very small sample sizes. A recent open consecutive cohort study demonstrated improvement in anxiety and depression symptoms of generalized anxiety order following 12 and 24 weeks of CES treatment with the Alpha-Stim AID device with bilateral earlobe electrodes (100 μA, 0.5 Hz); however, the study was not randomized or placebo-controlled, and the research group was funded by the manufacturer of the Alpha-Stim AIM device (Morriss et al., 2019; Morriss and Price, 2020).

One relatively large and well-conducted study shows compelling results for CES effectiveness in anxiety disorders (Barclay and Barclay, 2014). In this study, a group of 115 participants with a diagnosed anxiety disorder received either sham or active (100 μA, 0.5 Hz) CES for 5 weeks (60-min/day) using the Alpha-Stim 100 device with bilateral earlobe electrodes. The sham condition used inactive devices provided by the manufacturer, and no assessment of vestibular or cutaneous sensation was reported. By week 5, results demonstrated an ~32% reduction in anxiety symptoms measured using the Hamilton Rating Scale for anxiety (HAM-A-17). No long-term follow-up was reported.

### CES for Clinical Applications: Conclusions

In conclusion, about half of the relatively high-quality studies examining CES for insomnia treatment showed improvement in symptoms; these included improvement in latency to sleep onset, sleep quality, and sleep duration. It is important to note that while some studies showed no effects of CES, we did not find any studies suggesting a worsening of insomnia symptoms (Aseem and Hussain, 2019).

The lack of compelling evidence for CES effectiveness for mitigating depression symptoms likely underlies the FDA's decision to classify CES devices as Class III when marketed for the treatment of depression, given that the benefits do not clearly outweigh potential risks of adverse effects. The FDA therefore applies relatively stringent regulatory oversight for CES use in depression, in comparison to when marketed for treatment of insomnia or anxiety.

With clinical anxiety, we only identified one compelling study demonstrating beneficial effects of CES on the severity of anxiety symptoms, with most other studies showing methodological shortcomings and/or a high risk of bias. Again, while some studies showed no effects of CES on anxiety, we did not find any studies suggesting a worsening of symptoms with CES treatment.

Across all three categories of clinical application, we found very few rigorously designed experiments, with a pervasive lack of control for confounding variables (e.g., comorbidity, blinding, randomization, crossover). For example, most of the studies used an inactive sham control and did not assess possible vestibular or cutaneous sensation differences between active and sham groups that could be confounding results (Barclay and Barclay, 2014; McClure et al., 2015; Mischoulon et al., 2015; Wagenseil et al., 2018).

Even when well-controlled, experiments are generally inconclusive, showing inconsistently robust or reliable effects of CES on symptoms of insomnia, depression, or anxiety. There is also high methodological heterogeneity and lack of details, making it difficult to discern whether a study should be regarded as CES or tACS, *per se*. In our experience, CES is intended to provide relatively diffuse alternating current to the central and peripheral nervous systems by way of bilateral electrodes attached to the earlobes, temples, or orbital regions. In contrast, tACS uses electrode positions intended to target cortical regions more selectively, such as the prefrontal cortex or parietal lobe. As noted by other authors, there is generally a lack of compelling or consistent evidence for the effects of CES on clinical outcomes (Zaghi et al., 2010).

## CES For Non-Clinical Applications

With insomnia, depression, and anxiety, studies examining CES effects are conducted with clinical samples, many of whom were hospitalized, medicated, and/or show clinical comorbidities at the time of recruitment. This contrasts the potential application of CES technologies in healthy, non-clinical samples. Studies examining CES effects on healthy, neurotypical participants are relatively limited in number.

### CES for Acute Stress Mitigation

Three studies were identified examining CES effects on non-clinical state anxiety and stress. The first study involved 33 healthy participants undergoing routine dental procedures, randomly assigned to receive either active (0.5 Hz, 200 μA) or sham CES using the Alpha-Stim 100 with bilateral earlobe electrodes (Winick, 1999). In a double-blind design, participants received active or sham CES during a dental procedure, and reported symptoms of anxiety using a visual analog scale (VAS). The sham condition used an inactive device, and no assessments of vestibular or cutaneous sensation were reported between the active and sham groups. Results demonstrated that active CES produced significantly lower anxiety ratings post-treatment (but not during the treatment) relative to sham. The authors note, however, that there was an unequal distribution of dental procedure severity (e.g., routine cleaning vs. root canal) across treatment groups, potentially influencing the results.

A second study similarly examined CES in a dental setting, randomly assigning 40 participants to one of three multi-day interventions: relaxation therapy alone, CES alone (0.5 Hz, 100–600 μA) using the Alpha-Stim 100 device with bilateral earlobe electrodes, or the combination of the two (Koleoso et al., 2013). A fourth group served as a no-contact control, without any intervention, and no measures of vestibular or cutaneous sensation were collected in any group. Results showed that all three interventions reduced self-reported dental anxiety relative to control, but the three interventions did not differ from each another. In other words, CES did not provide any additional advantage for anxiety reduction relative to a relaxation therapy intervention. Note that participants were not blinded to treatment condition.

Another study examined CES for preoperative anxiety in 50 healthy women, prior to undergoing surgery for thyroidectomy. CES was administered using the Alpha-Stim 100 device with bilateral earlobe electrodes, using active (0.5 Hz, 100 μA) or sham stimulation immediately prior to surgery, in a single-blind, randomized, placebo-controlled design (Lee et al., 2013). The sham condition involved an inactive device, and no assessments of vestibular or cutaneous sensation differences across groups were reported. Results showed reduced anxiety and pain ratings in the CES vs. control groups, extending for at least 4 h post-surgery. Blood sampling showed no significant change in adrenocorticotrophic hormone (ACTH), cortisol, or glucose levels immediately prior to surgery; however, diurnal changes in cortisol, individual variability, and/or a low stress response to thyroidectomy may have interfered with the ability to identify any such effects.

### CES for Acute Sleep Quality Modification

A recent study examined the effects of CES on the sleep quality of 40 healthy women, in a randomized, sham-controlled (inactive device), double-blind study (Wagenseil et al., 2018). Using the Alpha-Stim 100 device with bilateral earlobe electrodes, active CES (0.5 Hz, 100 μA, for 60-min) was compared to a placebo control (device attached, but not active). Measures included polysomnography (PSG) and electroencephalography (EEG). PSG measures of sleep quality revealed no significant influence of CES. CES did induce a frequency-lowering effect in the alpha band of the EEG signal, similarly to what we will describe in the CES Effects on Electrical Brain Activity section.

### CES for Acute Human Performance Modification

In the only study examining cognitive performance outcomes of CES treatment in a healthy sample, 52 participants were randomly assigned to an active CES (15–500 Hz, intensity not disclosed, for 20-min) or sham (device attached, but not active) group in a double-blind design (Southworth, 1999). Stimulation was administered using the LISS Body Stimulator Bipolar Model, with bilateral electrodes placed below the temples. Participants completed a continuous performance test (CPT) at baseline, and then again following 20-min of active or sham stimulation. Results demonstrated improved attention (higher accuracy, faster response times) on the CPT following active vs. sham CES. No assessments of vestibular or cutaneous sensation were reported.

We found only one English-language study examining physical performance outcomes of CES treatment, involving 10 male weightlifters administered active CES (0.5 Hz, 10–500 μA, for 15-min) using the Alpha-Stim SCS device with bilateral earlobe electrodes (Cupriks et al., 2016). Before and after CES administration, muscle force output was measured during clean and press barbell lift repetitions (without the overhead phase). The authors reported that CES increased average and maximal force output. It is important to note, however, that this study was not placebo-controlled, randomized, or blinded in any manner. Furthermore, it is a very small sample size with specialized weightlifting skills.

### CES for Non-clinical Applications: Conclusions

In contrast to clinical applications, relatively few studies have examined CES effects on healthy, neurotypical participants. Among these, studies have emphasized CES effects on state anxiety responses, sleep quality, as well as cognitive and motor performance. In the three studies examining CES effects on anxiety, there was consistent support for CES reducing subjective feelings of anxiety, though this was not accompanied by expected endocrine response modulation. The fact that physiological indices of stress response were unchanged suggests that at least a portion of emotional responses seen with CES may be due to participant expectations and lack of effective blinding. In fact, all the identified studies used a sham procedure with a completely inactive device, increasing the risk that vestibular or cutaneous sensation differences between active and sham groups could be partially driving group differences. While authors tend to report verbal claims of subthreshold stimulation made by device manufacturers (Winick, 1999; Wagenseil et al., 2018), we found no quantitative evidence to support such a claim.

In the human performance domain, CES produced faster and more accurate performance on the CPT, and some cursory evidence for increased motor force output during a weightlifting task. The latter study is severely limited by its design, however, and results should be considered with caution. The effect of CES on CPT outcomes (Southworth, 1999) deserves continuing attention and replication. Any effects of CES on CPT must be disentangled from potential non-specific effects of neurostimulation; specifically, neuromodulatory techniques can cause intersensory facilitation, and arousal due solely to the sensory experiences of active stimulation (Campana et al., 2002; Dräger et al., 2004).

## CES Side Effects and Adverse Events

The most frequently reported side effects of CES administration are vertigo, skin irritation, and headaches (Kirsch and Nichols, 2013), which are estimated to occur about 1% of the time (Kirsch et al., 2014). In user manuals and reports published by device manufacturers, the guidance is to reduce stimulation intensity to mitigate any reported side effects; of course, in a research setting this strategy is undesirable due to differences in stimulation intensity across sessions or participants.

In studies not funded or published by authors associated with a CES device manufacturer, frequency of side effects is mixed. In one study using the Alpha-Stim SCS device with bilateral earlobe electrodes (0.5 Hz, 10–500 μA, 60-min), 25% (3/12) participants self-withdrew due to discomfort with side effects of dizziness or headache (Bystritsky et al., 2008). In another study using the Fisher Wallace Cranial Stimulator using a ramp-up sham procedure, side effect rates were generally high in both the active and sham conditions (e.g., 44% of participants reported headache) (McClure et al., 2015). Finally, another study using the Fisher Wallace Cranial Stimulator found high rates of poor concentration (59%) and malaise (29%) with active CES, both significantly greater than seen in the sham group (Mischoulon et al., 2015).

An early FDA-commissioned review of the safety of CES by the National Research Council (1974) stated, “significant side effects or complications attributable” to the application of electric current of ~1 mA or less for “therapeutic effect to the head” (i.e., cranial electrotherapy stimulation) were “virtually non-existent” (p. 42).

To examine more recent adverse events reported to the FDA by device users, we searched the FDA Manufacturer and User Facility Device Experience (MAUDE) database for records between 1990 and 2020 for the CES devices listed in the section titled A Brief History of CES. Three adverse reactions were reported during or following the use of an Alpha-Stim CES device, one in 2012 for burns experienced on earlobes, one in 2013 for onset of severe tinnitus, and one in 2019 for severe gastrointestinal distress and insomnia. Furthermore, seven adverse reactions were reported during or following the use of a Fisher Wallace CES device, including for disorientation, vestibular problems (balance, coordination, dizziness, vertigo), headaches, tinnitus, anxiety, depression, fatigue, brain hemorrhage, and death.

It is challenging to effectively dissociate side effects related to CES vs. comorbid health disorders, especially outside of the context of a controlled experiment. Given that some reported side effects may overlap with the symptoms of the disorder being treated, any worsening of symptoms should be taken seriously. For example, CES has been explored for the treatment of migraine and tension-related headaches while experimental reports of headache-related side effects of CES are also generally high (Solomon et al., 1989; Bystritsky et al., 2008; McClure et al., 2015).

## Mechanistic Explanations of CES Effects

**Figure 1:**
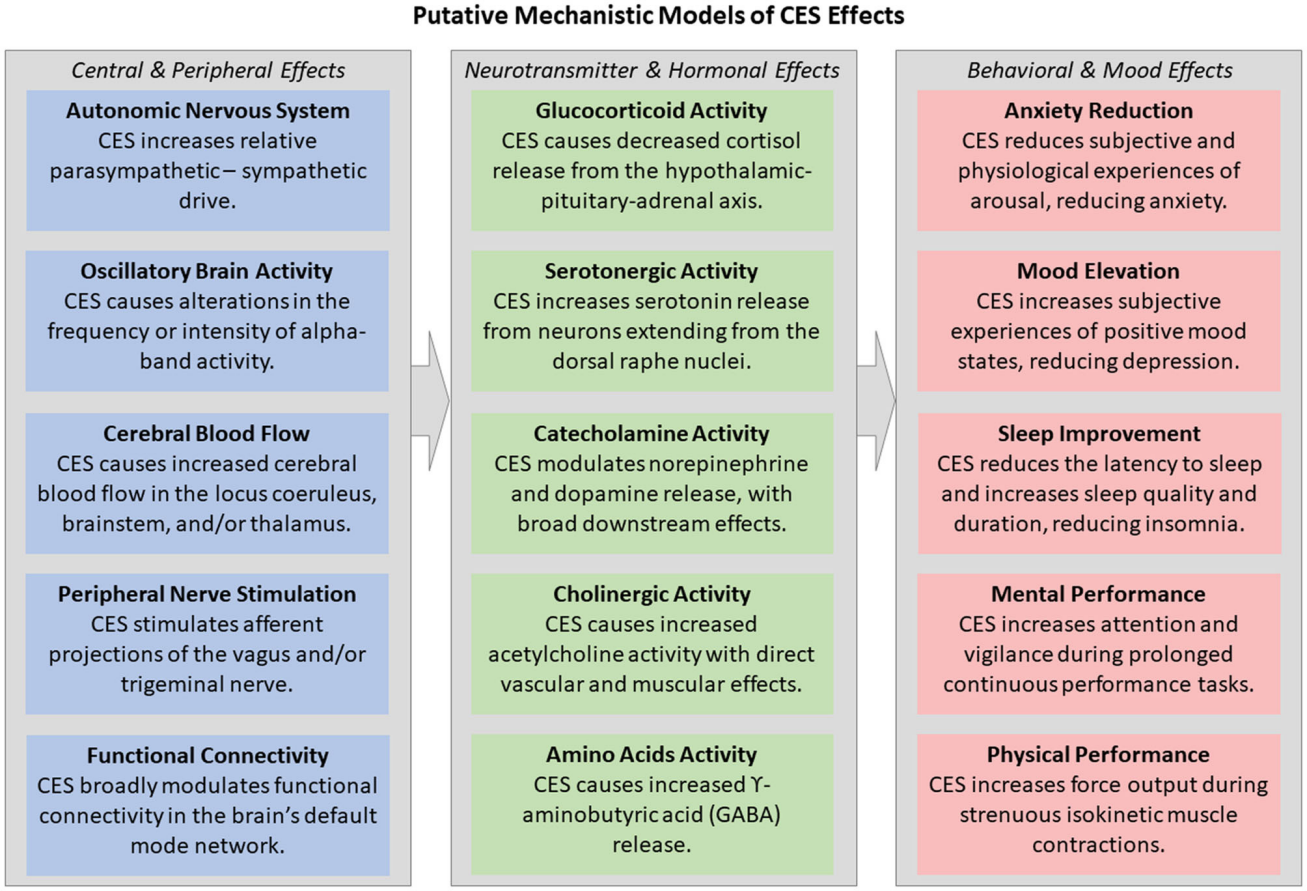
Past and present mechanistic explanations for CES effects on brain and behavior, including at the levels of the central and peripheral nervous system, neurotransmitters and hormones, and behavioral and mood effects.

Like other transcranial electrical stimulation (tES) methodologies, such as transcranial direct and alternating current stimulation (tDCS/tACS), the mechanisms underlying CES effects on brain and behavior remain elusive. This is for four primary reasons: a lack of well-controlled studies, equivocal experimental results, lack of methodological standardization, and frequent mischaracterization of study results (Edelmuth et al., 2010). Figure 1 demonstrates the heterogeneity of past and current mechanistic explanations for CES effects on the central and peripheral nervous system, neurotransmitters and hormones, and behavior and mood. Current computational modeling efforts and experimentation do suggest a mild effect of CES on brain activity (electrical, blood flow oxygenation) and potentially neurotransmitter and hormonal responses.

### Modeling CES Effects on the Central Nervous System

Computer-based modeling provides the opportunity to assess three critical questions regarding CES: does electrical current administered via CES reach cortical and/or subcortical brain regions, where are the effects of current propagation most and least pronounced, and how do morphological differences affect current flow across individuals? A few published papers explore these questions through computational modeling approaches.

The first model of CES current propagation predicted current density across four concentric anatomical spheres capturing the scalp, skull, cerebrospinal fluid, and cortical tissue (Ferdjallah et al., 1996). The authors were interested in whether applied electrical current might be dissipated over the surface of the scalp during stimulation, rendering little if any effect on underlying cortical tissue. The authors found that the maximum current density to reach relatively deep brain structures (e.g., thalamus) with a 1 mA intensity is ~5 μA/cm2 (15 V/m). This reported V/m value is exceedingly high and likely inaccurate, pointing to the need for more modern modeling tools. However, this model did provide the first evidence that at least a portion of electrical current administered via CES reaches cortical (and perhaps subcortical) brain regions. It did not, however, provide insights into the relative spatial distribution of current over various anatomical structures, or how morphological variation might impact current flow.

A second more realistic and comprehensive model of CES current propagation used a high-resolution head model (derived from magnetic resonance imaging; MRI) that predicts not only current density across cortical and subcortical targets, but also the effects of anatomical variation in gyri/sulci (Datta et al., 2013). The models considered 1.0 mA DC administration (at 150 Hz) with five different electrode montages, including conventional ear clips, and detailed current propagation across the scalp, skull, cerebrospinal fluid, eyes, muscle, gray and white matter, and air. With ear clips, peak current density ~0.10 V/m, with the strongest effects at the temporal regions and medulla oblongata, with diffuse effects across the midbrain, thalamus, pons, insula, and hypothalamus. An ear-hook montage induced the highest peak current intensity, at 0.47 V/m, with a relatively superior current flow through the cortex. Interestingly, with all electrodes, the current intensities reaching subcortical regions were similar to those reaching cortical regions, suggesting that CES may induce behavioral effects suggesting subcortical modulation (e.g., fatigue, attention, anxiety, sleep, appetite).

Thus, computational modeling of CES current propagation through tissue demonstrates that CES does effectively penetrate the scalp and skull, reaching both cortical and subcortical brain regions. The intensity of current at cortical and subcortical appear to be rather weak, though the two published models gave very mixed predictions (15 V/m vs. 0.47 V/m). Most CES current intensities predicted by computational modeling approaches are up to 1,000-times lower than the intensities induced with transcranial magnetic stimulation (TMS) (Bijsterbosch et al., 2012), predicting only subthreshold, if any, modulation of neuronal populations with CES. Furthermore, the second modeling effort may have limited applicability to CES given its emphasis on DC rather than AC stimulation.

### CES Effects on Electrical Brain Activity

Several studies have examined the effects of CES on electroencephalography (EEG) signals, using power spectral density analyses. EEG power spectral density refers to the frequency content of brain signals collected at the surface of the scalp. Frequency content of EEG signals is typically divided into at least four functionally distinct bands: Beta (12–40 Hz), Alpha (8–12 Hz), Theta (4–8 Hz), and Delta (1–4 Hz). The most common way to analyze activity in each frequency band is to calculate average band power, which aggregates the contribution of each frequency band to the overall power of the EEG signal (Tatum, 2014).

Power levels within each frequency band have been associated with physiological, cognitive, and affective processes (Niedermeyer and da Silva, 2005). Beta activity has been associated with motor planning, cognitive task engagement, alertness, anxiety, and rumination (Oken and Salinsky, 1992; Jacobs et al., 1996; Isotani et al., 2001). Alpha activity has been associated with idle motor behavior, eye closure, and relaxation (Niedermeyer, 1997; Feshchenko et al., 2001). Theta activity is relatively diminished in adulthood, showing most prominently in sleep-related phenomena such as near-sleep (hypnagogic state), rapid eye movement (REM) sleep, and sleep deprivation (Schacter, 1977). It has also been associated with attention, task engagement, memory, and cognitive performance (Klimesch, 1999). Delta activity has been associated with attention to internal thought processes, slow-wave sleep, homeostasis, motivation, salience detection, and subliminal perception (Harmony et al., 1996; Knyazev, 2012).

Given the associations between power in specific frequency bands and affective and cognitive functions, one might expect CES to decrease beta power, increase alpha power, and possibly decrease theta and/or delta power. Studies have found inconsistent support for these hypotheses. One study using the Alpha-Stim 100 device (0.5 or 100 Hz; 10–100 μA, 20-min) found no significant change in alpha band power with CES vs. sham, but a small shift to lower alpha frequencies overall during stimulation (Schroeder and Barr, 2001). In this study, the sham condition used an inactive device following a brief sensation thresholding procedure. However, this study used only a single EEG electrode for recording, examined only 12 male participants, and did not measure EEG after cessation of stimulation (introducing the possibility that some measured EEG effects were artifactual from CES administration). Another study described earlier in this report found a similar frequency shift in alpha band power with CES (Wagenseil et al., 2018). This latter study improved upon the earlier design, with 23-channel EEG (vs. 1-channel) recording, a larger sample size (40 vs. 12), and only measured EEG during a period in which the CES device was turned off.

A more recent study recorded EEG before and after CES administered with the Endomed 482 using bilateral earlobe electrodes at 0.5 or 100 Hz, with the sham group using an inactive device (no vestibular or cutaneous sensations were reported for either group). The authors found increased high frequency (11–13 Hz) parietal alpha band activity with 0.5 Hz stimulation, and increased low and mid-frequency temporal beta band activity with 100 Hz stimulation (Lee et al., 2019). The authors suggest these results show evidence for CES increasing “clear state of mind,” attention, and concentration. However, the study used a relatively low-density EEG montage (6 electrodes), did not reveal statistical outcomes for critical comparisons, and was not double-blinded.

Another study often cited by CES manufacturers in marketing materials was sourced to a student presentation at the International Society for Neuronal Regulation (Black et al., 2004). This pilot study was not randomized, placebo-controlled, or blinded, but the authors reported increased alpha band power following a single 20-min CES session (0.5 Hz, unknown intensity).

### CES Effects on Brain Hemodynamics

Two neuroimaging methods have been used to assess CES effects on brain hemodynamics, including magnetic resonance imaging (MRI) and Xenon-enhanced computed tomography (XeCT). The blood-oxygen-level-dependent (BOLD) signal is a primary measure of brain hemodynamics when using MRI, and is thought to index the extent to which blood is carrying oxygen to supply relatively active neurons (Raichle, 1998). In typical experiments, the BOLD signal is compared between multiple task conditions; for example, a cognitive task involving spatial orienting vs. inhibitory control. By comparing the spatial distribution of BOLD responses across the brain during performance of each task, researchers can derive insights into the brain regions that may underlie performance of one task vs. another.

In addition to the task-dependent BOLD response, MRI also provides measures of resting state activity across functionally connected regions of the brain. These networks of functional connectivity include the default mode network (DMN), sensorimotor network, frontoparietal network, and executive control network (Moussa et al., 2012). Activity in these and other networks has been used for a variety of clinical and neuropsychological purposes, including identifying and characterizing disease presence and prognosis (Greicius et al., 2004; Fox and Greicius, 2010), predicting cognitive task aptitudes (Hampson et al., 2006), and understanding the influence of clinical therapies on brain functional connectivity (Flodin et al., 2015; King et al., 2016).

Only one study has used MRI to evaluate effects of CES on brain hemodynamics. Eleven healthy participants were administered CES (0.5 and 100 Hz) for brief alternating periods of active and inactive stimulation (22-s each) using the Alpha-Stim 100 device with bilateral earlobe electrodes (Feusner et al., 2012). No control condition was used, though the authors did try to ensure that stimulation was subthreshold for cutaneous sensation in both the 0.5 and 100 Hz conditions. Measures of BOLD response demonstrated broad regional brain deactivation in both 0.5 and 100 Hz conditions, except for the thalamus. Measures of resting state network activity demonstrated that the 100 Hz condition was associated with alterations of DMN activity (both increased and decreased functional connectivity across nodes of the DMN), but not sensorimotor or frontoparietal networks. The authors proposed that the therapeutic effects of CES may be derived from a downregulation of internal thought related to worry or rumination, perhaps by shifting attention to external stimuli (Hamilton et al., 2011); however, it is important to note that CES did not produce any changes on the state-trait anxiety inventory (STAI). Methodological limitations include a small sample size, lack of a control condition, and no double-blinding.

Xenon-enhanced computed tomography (XeCT) uses CT scanning coupled with xenon gas inhalation by participants. The XeCT system measures the presence of the gas as it is carried by the blood and variably diffused into brain tissue, resulting in a quantitative measure of cerebral blood flow (CBF) (Zink, 2001). Only one study has used XeCT to evaluate CBF changes as a function of CES administration (Gense de Beaufort et al., 2012), using the Anesthelec device with three electrodes (one at center of forehead, two behind bilateral mastoids) and an inactive sham condition. In this study, the authors were interested in whether CES would modulate CBF in the brainstem and thalamus, given their role in pain and anxiety control, and links to the opioid system. A total of 36 healthy participants were randomly assigned to an active or sham CES group, and received a single 120-min stimulation following a baseline XeCT measurement. The device stimulated using a complex monophasic current at 100 Hz with reported peak-to-peak intensity of 280 mA, and XeCT was measured before and after CES administration. Results demonstrated no global change in CBF, but a pronounced decrease in CBF locally in the brainstem and thalamus, suggesting a role for CES impacts on these brain regions in modulating pain response and anxiety. No assessments of vestibular or cutaneous sensation differences were reported.

### CES Effects on Neurotransmitters and Hormones

Given the putative effects of CES on brain function, some studies have examined whether CES modulates salivary, urinary, cerebrospinal fluid, or blood levels of stress hormones (cortisol, alpha amylase, catecholamines, adrenocorticotropic hormone/ACTH), markers of inflammation and immune response (C-reactive protein, interleukin), and proteins reflecting the growth and survival of neurons (brain-derived neurotrophic factor/BDNF, nerve growth factor/NGF).

Some review articles claim evidence from animal models suggesting that CES can increase dopamine release in the basal ganglia in canines (Pozos et al., 1971), increase parasympathetic nervous system activation (Toriyama, 1975), or elevate β-endorphine levels in rat cerebrospinal fluid (Pert et al., 1981). However, these articles either describe a technique other than CES (e.g., electroacupuncture), or were not accessible for verification.

In humans, we found a total of five studies examining the effects of CES on neurotransmitter and/or hormone levels. In the first, 20 patients with alcoholism and affective disorders received either active (70–80 Hz, 4–7 mA) or control (≤ 1 mA constant current) stimulation for 4 weeks using an undisclosed device with three electrodes (one at center of forehead, two behind bilateral mastoids), in a double-blind placebo-controlled study (Krupitsky et al., 1991). No assessments of vestibular or cutaneous sensations were reported. In comparing the groups post-treatment, the authors found increased blood levels of monoamine oxidase-B (MAO-B) and gamma aminobutyric acid (GABA), but no change in levels of serotonin, dopamine, or β-endorphins.

In a second study, 52 cancer patients received active (0.5 Hz, 0.1 mA) stimulation using the Alpha-Stim M device for 4 weeks (with bilateral earlobe electrodes, used for 60-min/day) (Yennurajalingam et al., 2018). There was no control condition, and no assessments of vestibular or cutaneous sensation were reported. When comparing baseline vs. after 4-weeks of CES, no differences were found in salivary measures of alpha amylase, cortisol, C-reactive protein, interleukin-1, or interleukin-6. These results suggest that CES treatment, at least with these methodological parameters, does not reliably modulate stress hormones or markers of inflammation and immune response.

In a third study, 36 obese females were randomly assigned to one of three groups: aerobic exercise only, aerobic exercise and CES, or control (Cho et al., 2016). Active CES was administered with the Alpha-Stim 100 device with bilateral earlobe electrodes for 20-min at 100 μA intensity and 0.5 Hz; the control group was no-contact without any sham procedure, and no measures of vestibular or cutaneous sensation were reported. While serum BDNF and NGF increased after all interventions, they did not differ between the exercise only vs. exercise and CES groups. Similar results were found with cortisol and ACTH, with no significant differences between those two groups.

In a fourth study, 15 participants (note: including the authors and colleagues) received active or sham CES for 20-min with one of two waveforms (1 mA at 15 Hz bipolar or monopolar) delivered by the LISS Cranial Stimulator with electrodes placed “transcranially” at undisclosed locations (Liss and Liss, 1996). The sham condition involved an inactive device, and no measures of vestibular or cutaneous sensation were reported. The authors found evidence that CES treatment increased ACTH, β-endorphine, and serotonin levels, and decreased cortisol levels. It is important to note that in addition to the experimenters testing themselves, additional competing interest and methodological limitations were present, including the authors' proprietorship of a CES manufacturer, and the lack of blinding or random assignment.

Finally, a more recent and better controlled study examined 50 healthy women randomly assigned to active CES (0.5 Hz, 100 μA, 20-min/day for 8 weeks) or sham (device attached, but inactive) using the Alpha-Stim 100 device with bilateral earlobe electrodes, in an open label design (Roh and So, 2017). Blood samples were collected to assess cortisol, ACTH, BDNF, and NGF, and the profile of mood states (POMS) was administered to measure subjective anxiety. The authors found evidence that CES reduced anxiety ratings on the POMS, but did not significantly alter any blood levels of neurotrophic factors or hormones. These results were similar to those found by Lee et al. (2013), as discussed previously.

Note also that some other articles frequently cited in reviews as support for CES effects on neurotransmitters or hormones were limited in various ways; for example, using transcutaneous electrical stimulation of the legs or arms rather than cranial stimulation (Salar et al., 1981), or using a sham CES procedure that is active and a higher intensity than active CES used in other published papers (Gabis et al., 2003).

Overall, only one study suggested CES effects on neurotransmitter activity (Liss and Liss, 1996), but it suffers from high risk of bias and conflict of interest.

### CES Effects on Parasympathetic Nervous System Activity

One model of CES action on the brain and behavior (Gilula, 2007) suggests very broad and diverse neuromodulatory effects across the limbic system, reticular-activating system, and thalamus and hypothalamus. This network of modulation predicts changes in sensory processing, regulation of mood states, altered arousal states, and even analgesia, perhaps through activation of the parasympathetic division of the autonomic nervous system.

Such diverse neuromodulatory effects may arise from stimulating afferent projections of peripheral nerves such as the trigeminal nerve, vagus nerve, facial nerve, and/or auditory nerve. Indeed, one proposed effect of CES is increased parasympathetic nervous system activation due to stimulation of the vagus nerve, with or without any cortical modulation (Zaghi et al., 2010; Howland, 2014; Asamoah et al., 2019). While this is a compelling possibility, there is no direct evidence for CES effects on relative parasympathetic/sympathetic activity.

## Methodological Limitations of CES Research

Through our review, we have identified several limitations to the extant literature examining CES effects on clinical and non-clinical participants. An overview of these limitations is below.

### Potential Conflicts of Interest

Conflicts of interest (COI) occur when professional judgments or actions regarding a primary interest (e.g., sound research) are influenced by a secondary interest (e.g., financial gain) (Lo and Field, 2009). For example, a primary interest to conduct research in a sound, methodical, and honest manner may be unduly influenced by a secondary interest of financial gain, promotion, or recognition. Conflicts of interest can arise when an investigator is also a patient's physician, and/or when they stand to benefit from the success of an investigated drug or therapy.

There is a long history of potential COI in CES research. Nearly half of the published CES research we found appears to be funded by CES manufacturers or authored by the founders, owners, management, consultants, or board members of CES manufacturers or retailers. These authors stand to benefit from positive effects of CES systems, introducing the possibility that results are influenced (intentionally or unintentionally) by the potential COI. These COIs may cause authors to aggregate results in reviews or meta-analyses in a biased manner or misrepresent the design or results of primary research. Examples include omitting primary research that does not show positive benefits of CES, and citing primary research that did not examine CES specifically, examined animal models rather than humans, or was not published in a peer-reviewed journal (e.g., student poster presentations). As noted by other reviews on this topic, many reviews authored by individuals associated with CES device manufacturers “did not report any formalized search strategy, inclusion criteria or quality assessment and discussed a number of unpublished studies that remain unpublished at the time of the current review” (O'Connell et al., 2011). Together, it is our impression that a large portion of the existing CES literature has high potential for COI influencing data, theory, and application.

### Risk of Bias in Clinical Trials

The Cochrane Collaboration has developed guidelines and software tools for assessing the risk of bias in clinical trials (Higgins et al., 2011; Sterne et al., 2019). Five domains of bias are identified: bias arising from the randomization process, bias due to deviations from intended interventions, bias due to missing outcome data, bias in measurement of the reported outcome, and bias in selection of the reported result. In our review of the CES literature, we found several examples of these potential biases. Many studies did not use (or did not report) random selection, had missing outcome data due to data loss or participant attrition, used non-standardized outcome measures with high subjectivity, and/or did not comprehensively report all results. For these reasons, reviews of CES effects conforming to the Cochrane guidelines are severely limited in the number of studies that can be included in a review.

For example, in a review and meta-analysis of non-invasive brain stimulation (NIBS) effects on chronic pain, applying the Cochrane guidelines resulted no single CES study being judged as having a low risk of bias (O'Connell et al., 2011). Even when including potentially biased studies, the meta-analysis demonstrated no significant advantage of CES relative to placebo. Similarly, a Cochrane review of CES for headache therapy revealed only one study that fit Cochrane criteria, though baseline group differences limited results interpretation (Brønfort et al., 2014).

In a meta-analysis of CES effects on depression (and headache, insomnia, anxiety, and brain dysfunction) co-authored by the chairman of a CES device manufacturer (Kirsch and Gilula, 2007), we encountered major challenges interpreting results due to four primary weaknesses: first, the authors aggregated data from both open label and blinded studies; second, the analysis included studies showing very high comorbidity of disorders, including fibromyalgia, insomnia, anxiety, alcoholism, and attention disorders; third, no formal inclusion or exclusion criteria were provided for their literature search; fourth, the authors combined a highly heterogenous set of clinical ratings scales into a single percent improvement score, and did not compare it to a sham group (Kavirajan et al., 2014). Interestingly, the authors also selectively excluded a study that showed a negative influence of CES, suggesting that it was not valid due to improvement in the sham condition.

A similar monograph, including a review and informal meta-analysis, was published by an author affiliated with a different CES device manufacturer (Smith, 2007). This report suffers from similar weaknesses to the one cited above (Kavirajan et al., 2014). Interestingly, this author also offers [on a device manufacturers website; (Smith, 2013)] to analyze data and prepare manuscripts related to CES effects free of charge. While we did not find any statements disclosing Smith's relationships with CES device companies, we did find evidence that he was (and perhaps remains) the Director of Science for Electromedical Products International, Inc. (Kirsch and Smith, 2000).

Finally in a more recent, well-conducted review and meta-analysis of CES effects on depression using the Cochrane guidelines, not a single study passed the Cochrane criteria for inclusion in the meta-analysis (Kavirajan et al., 2014).

### Sham Credibility and Blinding

Active CES commonly causes feelings of dizziness, cutaneous tingling or skin irritation at the electrode sites, or light-headedness (Bystritsky et al., 2008; Amr et al., 2013; Kirsch and Nichols, 2013; Wu et al., 2020). In fact, some studies use dizziness or light-headedness as a criterion for identifying the appropriate stimulation intensity for an individual participant; specifically, increasing stimulation intensity until the participant reports these symptoms, and then reducing intensity slightly from that threshold (Bystritsky et al., 2008; Kirsch and Chan, 2013). Of course, any sensory habituation achieved during this phase of a study could be changed or eliminated during subsequent phases or sessions. Most studies reviewed here, however, report a static AC frequency and intensity for active CES administration, rather than customizing CES intensity to individual tolerances; this is especially the case for non-clinical settings. In our own testing of the Alpha-Stim M, we found suprathreshold cutaneous sensation and vertigo at or above 100 μA (0.5 Hz). We are currently conducting a double-blind, cross-over study to assess cutaneous and vestibular sensations induced via CES at various intensities and frequencies. The possibility that users can readily identify when the device is active vs. inactive increases the likelihood that any attempts at participant blinding to CES conditions will be ineffective, particularly in cross-over designs (O'Connell et al., 2011).

Relatedly, in our review of the literature we found a highly mixed application of sham procedures. In most cases, the CES electrodes were attached to the participant, but the device was never turned on (Liss and Liss, 1996; Schroeder and Barr, 2001; Gense de Beaufort et al., 2012; Roh and So, 2017). In some other cases, there was either no control group (Feusner et al., 2012; Yennurajalingam et al., 2018), a no-contract control group (Cho et al., 2016), or the device was active but at a lower intensity than in the active CES condition (Krupitsky et al., 1991; Wu et al., 2020). The latter procedure is intended to produce mild cutaneous sensations in both groups, reducing the likelihood that participants can determine whether they are receiving active or sham CES. However, it should be noted that some low-intensity sham procedures use a higher intensity (e.g., 0.75 mA) stimulation than the active CES used in other studies (Gabis et al., 2003, 2009; O'Connell et al., 2011). In other words, these low-intensity sham procedures could be inducing similar effects to the active procedures used in other studies, reducing the likelihood of finding effects across treatment groups, and limiting comparability to other studies.

### Parameter Heterogeneity

Across published studies, CES is administered using a variety of parameters, including the number, type, and placement of electrodes, the timing and duration of stimulation, and the amplitude, intensity, and dynamics of AC waveforms. Stimulation electrodes are typically placed on the temples, mastoids, and/or ear lobes, and vary in size between small ear lobe clips and large saline-soaked sponges (Zaghi et al., 2010; Datta et al., 2013). As reviewed by Zaghi, stimulation parameters typically vary in duration from between 5 and 30 min, and intensity between 0.1 and 4.0 mA (Zaghi et al., 2010). The timing of CES administration relative to outcome measures, and the frequency and duration of stimulation also vary dramatically across studies.

Alternating current dynamically alternates polarity over time, typically represented by sinusoidal waveform. With CES, device manufacturers typically modify the amplitude, frequency, and shape of the waveform. In terms of amplitude, we found studies using amplitudes as low as 100 μA (Lande and Gragnani, 2013; Wagenseil et al., 2018) and as high as 4 mA (Mischoulon et al., 2015), with one outlying study reporting the use of a novel Limoge waveform at 49 mA (Gense de Beaufort et al., 2012). For frequency, we found studies using AC frequencies reported as low as 0.5 Hz (Winick, 1999; Koleoso et al., 2013; Cho et al., 2016) and as high as 15,000 Hz (Southworth, 1999; McClure et al., 2015; Mischoulon et al., 2015). The shape of the waveforms also vary dramatically across studies and devices, including symmetrical or asymmetrical biphasic waveforms, unmodified and modified square waveforms, and monophasic and biphasic waveforms (O'Connell et al., 2011; Bikson et al., 2019).

The heterogeneity of stimulation methods, including electrode types and placement, stimulation timing and parameters, all influence the reproducibility, comparability, and generalizability of research outcomes in CES, making it challenging to derive insights into its suitability for application in clinical or non-clinical domains. These issues are compounded by the fact that many of the devices used in past research are obsolete, antiquated, and otherwise unavailable for scientists to conduct replication attempts.

## Future Research Directions

We propose two primary directions for continuing CES research, particularly regarding non-clinical applications. First is basic research characterizing CES effects on central and peripheral nervous system activity and behavior, with potential application to modulating stress responses and possibly mitigating adverse performance effects under conditions of stress. Second is more attention to methodological considerations in the design, analysis, and reporting of CES research. We consider these two topics, in turn.

Threats to the physical or social self, uncertainty and novelty, and the perceived uncontrollability of situations all produce transient stress states (Mason, 1968). Adaptability under conditions of stress and uncertainty is critical to sustaining cognitive performance, and maladaptive responses under these circumstances give rise to long-term negative repercussions for psychological well-being (Grupe and Nitschke, 2013). Acute stress causes reliable physiological, affective, and cognitive responses. Physiologically, stress activates the sympathetic nervous system and causes a rapid release of catecholamines, namely epinephrine and norepinephrine. A second, slower response is activation of the hypothalamic-pituitary-adrenal (HPA) axis, resulting in the release of corticotropin-releasing hormone (CRH), ACTH, and cortisol. Activating these two stress systems produces a cascade of hormonal and neural effects throughout the central and peripheral nervous systems, with downstream effects on perception, affective states, and cognition (Gagnon and Wagner, 2016).

Stress is often seen as adaptive (Charmandari et al., 2005; Grupe and Nitschke, 2013) and can help direct selective attention and increase vigilance to sensory input (Eysenck et al., 2007; Pessoa, 2009). However, stress can also induce impairments in tasks involving several brain structures sensitive to the presence of catecholamines and glucocorticoids, including the prefrontal cortex, hippocampus, striatum, and amygdala (Arnsten, 1998; Hermans et al., 2014; Kim et al., 2015). These brain regions play diverse roles in our ability to attend to, process, understand, and use information in an adaptive manner. Indeed acute stress can degrade performance on working memory tasks (Luethi et al., 2008), disrupt visuomotor task performance and top-down attentional control (Vedhara et al., 2000; Vine et al., 2016), impair memory encoding and/or retrieval (Gagnon and Wagner, 2016), and impair cognitive control and flexible, goal-directed thinking in general (Eysenck et al., 2007).

Given the diverse and reliable effects of acute stress on the brain, cognition, and behavior, candidate technologies or methodologies to temporarily reduce the intensity or duration of the stress response are of interest to the defense science community. Candidate technologies could be used during training, in real-time during stressful occupational tasks, or to help facilitate recovery post-stressor.

CES is one candidate technology that could hold potential in reducing the downstream hormonal, neural, and behavioral effects of stress by modulating central and peripheral nervous system activity. Specifically, a transient increase in relative parasympathetic activation (i.e., altering the sympathetic/parasympathetic balance), whether through cranial (vagus) nerve stimulation or *n*th order neuromodulatory effects of CES, would carry effects for several brain regions, including the thalamus, hypothalamus, amygdala, locus coeruleus, cerebellum, orbitofrontal cortex, and medulla (Chae et al., 2003). A reduction in sympathetic drive would increase relative parasympathetic dominance (Clancy et al., 2014), possibly leading to reductions in inflammatory responses (Borovikova et al., 2000; Breit et al., 2018), and altering levels of hormones, peptides and neurotransmitters such as norepinephrine, serotonin, and cortisol. Further downstream effects of vagus stimulation would include alteration of activity in at least the heart, respiratory system, stomach (including small and large intestine), liver, and pancreas (Breit et al., 2018). These possibilities point to future basic research directions with CES.

We also make five primary methodological recommendations for continuing CES research. First, we suggest using larger sample sizes to increase power and the likelihood of identifying any true effects that may exist. Many CES studies have very small sample sizes that lower power and increase the likelihood of Type I errors; small sample sizes and are unlikely to identify a true effect, hold low predictive value, and any identified effects are likely inflated (Button et al., 2013). To justify small sample sizes, some papers cite existing research also with small sample sizes but showing strong effect sizes; however, to better estimate sample size needs, authors may find value in using meta-analytic estimates of effect size, rather than single effect sizes found in studies that may also suffer from methodological challenges.

Second, assuming sample size criteria are adequately defined and met, scientists, institutions, and publishers should assign equal value to manuscripts reporting null or unexpected results (Schooler, 2011; Martin and Clarke, 2017). Publication bias toward positive findings occurs not only in original science, but also in replication attempts and contaminates theory development and the systematic aggregation of results via meta-analysis (Francis, 2012). Third, registered reports are an effective tool for reducing publication bias and increasing the transparency and reproducibility of science (Schooler, 2011). In these cases, authors propose their complete methodology and analysis plan and receive in-principle acceptance for the manuscript, regardless of whether data ultimately support their hypotheses.

Fourth, parameter standardization across studies and laboratories will help researchers and clinicians derive more reliable understandings of how CES electrode placement, stimulation frequency, waveform, intensity and duration, all influence clinical and human performance outcomes with CES. Standardizing parameter selection and manipulation will inform the most reliable and robust ways to administer CES, and also facilitate predictive modeling efforts in this regard. Finally, while it may be appealing to reduce cost or increase research efficiencies by partnering with individuals or corporations with conflicting interests, ultimately these relationships can limit progress in research and clinical practice. Independently supported research using sound methodologies and analytical and reporting techniques will only benefit the research community and populations interested in using these tools to reduce symptoms of clinical disorders.

## Conclusions

CES was developed as a tool for treating the symptoms of clinical disorders such as insomnia, anxiety, and depression. The FDA differently regulates CES devices based on their intent, with relatively stringent controls (Class III) for the treatment of depression, given a lack of data demonstrating that any benefits outweigh potential risks. Studies examining CES effectiveness in the treatment of these disorders are equivocal, and there is generally a lack of compelling evidence from well-designed studies. Notably, however, no single study showed a worsening of insomnia, anxiety, or depression symptoms during or following CES treatment. It is worth considering that any placebo effects that may be elicited by CES due to participant expectations, experimenter bias, and/or cutaneous or vestibular perception may be sufficient enough to induce behavioral and physiological effects (Enserink, 1999; Dräger et al., 2004).

Very few studies have examined CES for application to healthy, neurotypical populations. In studies examining acute stress responses in healthy participants, CES appears to reduce subjective feelings of anxiety, but these are not necessarily accompanied by any changes in endocrine responses. In two human performance-oriented studies, CES produced faster and more accurate responding on certain CPT measures (Southworth, 1999), and increased motor force output during a weightlifting task (Cupriks et al., 2016). The former outcome warrants replication and extension, whereas the latter study has considerable methodological challenges that limit interpretation. Reproducible, reliable (within and across participants), and robust demonstrations of CES effects on nervous system activity and behavior are necessary before adopting CES for use in occupational contexts, including training, job performance, and recovery contexts (Bostrom and Sandberg, 2009; Agar, 2013; Chatterjee, 2013; Colzato, 2018; Dessy et al., 2018; Blacker et al., 2019; Feltman et al., 2019; Brunyé et al., 2020).

Several studies have attempted to elucidate the mechanisms underlying CES effects on brain and behavior. Among these are studies examining: (1) computational modeling of current propagation through the skin, skull, cerebrospinal fluid, and brain, (2) CES effects on electrical brain activity and hemodynamics, and (3) CES effects on endocrine and neurotransmitter systems. In general, computational modeling efforts predict CES currents effectively penetrating the scalp and scull and reaching cortical and subcortical brain regions, however at very low current intensities at target. Brain monitoring studies have shown inconsistent support for changes in frequency band activity using EEG, but preliminary support for changes in default mode network activity and reduced activity in brain stem and limbic systems. Finally, studies examining CES effects on neurotransmitters and hormones are very mixed, with most finding no evidence that CES modulates markers of inflammation, immune response, or stress hormones.

Most existing CES research suffers from considerable methodological challenges. The primary ones identified were potential conflicts of interest, risk of bias, sham credibility and blinding, and the heterogeneity of CES parameters used (Kavirajan et al., 2014; Shekelle et al., 2018a). These limitations make it difficult to derive consistent or compelling insights from the extant literature, tempering our enthusiasm for CES and its potential to alter brain function, behavior, or endocrine responses reliably or robustly, in clinical or non-clinical settings.

The lack of compelling evidence also motivates well-designed and relatively high-powered experiments to assess how CES might modulate the physiological, affective, and cognitive responses to stress. Indeed, the challenges faced by CES research are similar to those faced by other contentious research topics in psychology and neuroscience (Earp and Trafimow, 2015; Maxwell et al., 2015), including other domains of neurostimulation (Koenigs et al., 2009; Brem et al., 2014; Horvath et al., 2014; Vannorsdall et al., 2016; Brunyé et al., 2018), and would likely benefit from similar methodological and reporting improvements. Continuing transparency and methodological improvements with CES will allow the scientific community to develop more informed and nuanced understandings of how CES can be used to modulate nervous system activity and behavior, with potentially expanded applications to both clinical and non-clinical settings.

## Author Contributions

This report was drafted by TB. Revised and expanded upon by JP, TW, and EH. All authors contributed to the article and approved the submitted version.

## Conflict of Interest

The authors declare that the research was conducted in the absence of any commercial or financial relationships that could be construed as a potential conflict of interest.

## References

[B1] Agar N. (2013). Truly Human Enhancement: A Philosophical Defense of Limits. MIT Press. 10.7551/mitpress/9780262026635.001.0001

[B2] Amr M. El-Wasify M. Elmaadawi A. Z. Roberts R. J. El-Mallakh R. S. (2013). Cranial electrotherapy stimulation for the treatment of chronically symptomatic bipolar patients. J. ECT 29, e31–32. 10.1097/YCT.0b013e31828a344d 23670021

[B3] Arnsten A. F. T. (1998). Catecholamine modulation of prefrontal cortical cognitive function. Trends Cogn. Sci. 2, 436–447. 10.1016/S1364-6613(98)01240-6 21227275

[B4] Asamoah B. Khatoun A. Mc Laughlin M. (2019). TACS motor system effects can be caused by transcutaneous stimulation of peripheral nerves. Nat. Commun. 10:266. 10.1038/s41467-018-08183-w 30655523 PMC6336776

[B5] Aseem A. Hussain M. E. (2019). Impact of cranial electrostimulation on sleep: a systematic review. Sleep Vigilance 3, 101–112. 10.1007/s41782-019-00075-3

[B6] Barclay T. H. Barclay R. D. (2014). A clinical trial of cranial electrotherapy stimulation for anxiety and comorbid depression. J. Affect. Disord. 164, 171–177. 10.1016/j.jad.2014.04.029 24856571

[B7] Bijsterbosch J. D. Barker A. T. Lee K. H. Woodruff P. W. R. (2012). Where does transcranial magnetic stimulation (TMS) stimulate? Modelling of induced field maps for some common cortical and cerebellar targets. Med. Biol. Eng. Comput. 50, 671–681. 10.1007/s11517-012-0922-8 22678596

[B8] Bikson M. Esmaeilpour Z. Adair D. Kronberg G. Tyler W. J. Antal A.. (2019). Transcranial electrical stimulation nomenclature. Brain Stimul. 12, 1349–1366. 10.1016/j.brs.2019.07.010 31358456 PMC6851475

[B9] Black L. Cannon R. Hanslmayr S. Kennerly R. Rothove J. Sherlin L. (2004). Student Scholarship Presentation Abstracts. J. Neurother. 8, 107–118. 10.1300/J184v08n02_11

[B10] Blacker K. J. Hamilton J. Roush G. Pettijohn K. A. Biggs A. T. (2019). Cognitive training for military application: a review of the literature and practical guide. J. Cogn. Enhanc. 3, 30–51. 10.1007/s41465-018-0076-1

[B11] Borovikova L. V. Ivanova S. Zhang M. Yang H. Botchkina G. I. Watkins L. R.. (2000). Vagus nerve stimulation attenuates the systemic inflammatory response to endotoxin. Nature 405, 458–462. 10.1038/35013070 10839541

[B12] Bostrom N. Sandberg A. (2009). Cognitive enhancement: Methods, ethics, regulatory challenges. Sci. Eng. Ethics 15, 311–341. 10.1007/s11948-009-9142-5 19543814

[B13] Bracciano A. G. Chang W. P. Kokesh S. Martinez A. Meier M. Moore K. (2012). Cranial electrotherapy stimulation in the treatment of posttraumatic stress disorder: a pilot study of two military veterans. J. Neurother. 16, 60–69. 10.1080/10874208.2012.650100

[B14] Breit S. Kupferberg A. Rogler G. Hasler G. (2018). Vagus nerve as modulator of the brain–gut axis in psychiatric and inflammatory disorders. Front. Psychiatry 9:44. 10.3389/fpsyt.2018.00044 29593576 PMC5859128

[B15] Brem A. K. Fried P. J. Horvath J. C. Robertson E. M. Pascual-Leone A. (2014). Is neuroenhancement by noninvasive brain stimulation a net zero-sum proposition? Neuroimage 85, 1058–1068. 10.1016/j.neuroimage.2013.07.038 23880500 PMC4392930

[B16] Brønfort G. Haas M. Evans R. L. Goldsmith C. H. Assendelft W. J. Bouter L. M. (2014). Non-invasive physical treatments for chronic/recurrent headache. Cochrane Database Syst. Rev. 2014:CD001878. 10.1002/14651858.CD001878.pub3 25157618 PMC6483320

[B17] Brunyé T. T. Brou R. Doty T. J. Gregory F. D. Hussey E. K. Lieberman H. R. (2020). A Review of US army research contributing to cognitive enhancement in military contexts. J. Cogn. Enhanc. 4, 453–468. 10.1007/s41465-020-00167-3

[B18] Brunyé T. T. Smith A. M. Horner C. B. Thomas A. K. (2018). Verbal long-term memory is enhanced by retrieval practice but impaired by prefrontal direct current stimulation. Brain Cogn. 128, 80–88. 10.1016/j.bandc.2018.09.008 30414699

[B19] Button K. S. Ioannidis J. P. A. Mokrysz C. Nosek B. A. Flint J. Robinson E. S. J.. (2013). Power failure: why small sample size undermines the reliability of neuroscience. Nat. Rev. Neurosci. 14, 365–376. 10.1038/nrn3475 23571845

[B20] Bystritsky A. Kerwin L. Feusner J. (2008). A pilot study of cranial electrotherapy stimulation for generalized anxiety disorder. J. Clin. Psychiatry 69, 412–417. 10.4088/JCP.v69n0311 18348596

[B21] Campana G. Cowey A. Walsh V. (2002). Priming of motion direction and area V5/MT: a test of perceptual memory. Cerebral Cortex 12, 663–669. 10.1093/cercor/12.6.663 12003865

[B22] Chae J. H. Nahas Z. Lomarev M. Denslow S. Lorberbaum J. P. Bohning D. E.. (2003). A review of functional neuroimaging studies of vagus nerve stimulation (VNS). J. Psychiatr. Res. 37, 443–455. 10.1016/S0022-3956(03)00074-8 14563375

[B23] Charmandari E. Tsigos C. Chrousos G. (2005). Endocrinology of the stress response. Annu. Rev. Physiol. 67, 259–284. 10.1146/annurev.physiol.67.040403.120816 15709959

[B24] Chatterjee A. (2013). Chapter 27—The ethics of neuroenhancement, in Handbook of Clinical Neurology, eds J. L. Bernat and H. R. Beresford (New York, NY: Elsevier), 323–334. 10.1016/B978-0-444-53501-6.00027-5 24182389

[B25] Cho S. Y. So W. Y. Roh H. T. (2016). Effects of aerobic exercise training and cranial electrotherapy stimulation on the stress-related hormone, the neurotrophic factor, and mood states in obese middle-aged women: a pilot clinical trial. Salud Mental 39, 249–256. 10.17711/SM.0185-3325.2016.029

[B26] Clancy J. A. Mary D. A. Witte K. K. Greenwood J. P. Deuchars S. A. Deuchars J. (2014). Non-invasive vagus nerve stimulation in healthy humans reduces sympathetic nerve activity. Brain Stimul. 7, 871–877. 10.1016/j.brs.2014.07.031 25164906

[B27] Colzato L. S. (2018). Responsible cognitive enhancement: neuroethical considerations. J. Cogn. Enhanc. 2, 331–334. 10.1007/s41465-018-0090-3

[B28] Cupriks L. Vimbsons V. Cuprika A. Rudzitis A. (2016). Cranial electrical stimulation in fitness with weightlifting tools. LASE J. Sport Sci. 7, 21–32. 10.1515/ljss-2016-0010

[B29] Datta A. Dmochowski J. P. Guleyupoglu B. Bikson M. Fregni F. (2013). Cranial electrotherapy stimulation and transcranial pulsed current stimulation: a computer based high-resolution modeling study. Neuroimage 65, 280–287. 10.1016/j.neuroimage.2012.09.062 23041337

[B30] Dessy E. Van Puyvelde M. Mairesse O. Neyt X. Pattyn N. (2018). Cognitive performance enhancement: do biofeedback and neurofeedback work? J. Cogn. Enhanc. 2, 12–42. 10.1007/s41465-017-0039-y

[B31] Dräger B. Breitenstein C. Helmke U. Kamping S. Knecht S. (2004). Specific and nonspecific effects of transcranial magnetic stimulation on picture-word verification. Eur. J. Neurosci. 20, 1681–1687. 10.1111/j.1460-9568.2004.03623.x 15355336

[B32] Earp B. D. Trafimow D. (2015). Replication, falsification, and the crisis of confidence in social psychology. Front. Psychol. 6:21. 10.3389/fpsyg.2015.00621 26042061 PMC4436798

[B33] Edelmuth R. C. Nitsche M. A. Battistella L. Fregni F. (2010). Why do some promising brain-stimulation devices fail the next steps of clinical development? Exp. Rev. Med. Dev. 7, 67–97. 10.1586/erd.09.64 20021241

[B34] Enserink M. (1999). Can the placebo be the cure? Science 284, 238–240. 10.1126/science.284.5412.238 10232967

[B35] Eysenck M. W. Derakshan N. Santos R. Calvo M. G. (2007). Anxiety and cognitive performance: attentional control theory. Emotion 7, 336–353. 10.1037/1528-3542.7.2.336 17516812

[B36] Feighner J. P. Brown S. L. Olivier J. E. (1973). Electrosleep therapy: a controlled double blind study. J. Nerv. Ment. Dis. 157, 121–128. 10.1097/00005053-197308000-00004 4724809

[B37] Feltman K. A. Hayes A. M. Bernhardt K. A. Nwala E. Kelley A. M. (2019). Viability of tDCS in military environments for performance enhancement: a systematic review. Mil. Med. 185, e53–e60. 10.1093/milmed/usz189 31735955

[B38] Ferdjallah M. Bostick F. X. Barr R. E. (1996). Potential and current density distributions of cranial electrotherapy stimulation (CES) in a four-concentric-spheres model. IEEE Trans. Biomed. Eng. 43, 939–943. 10.1109/10.532128 9214809

[B39] Feshchenko V. A. Reinsel R. A. Veselis R. A. (2001). Multiplicity of the alpha rhythm in normal humans. J. Clin. Neurophysiol. 18, 331–344. 10.1097/00004691-200107000-00005 11673699

[B40] Feusner J. D. Madsen S. Moody T. D. Bohon C. Hembacher E. Bookheimer S. Y.. (2012). Effects of cranial electrotherapy stimulation on resting state brain activity. Brain Behav. 2, 211–220. 10.1002/brb3.45 22741094 PMC3381625

[B41] Flodin P. Martinsen S. Mannerkorpi K. Löfgren M. Bileviciute-Ljungar I. Kosek E.. (2015). Normalization of aberrant resting state functional connectivity in fibromyalgia patients following a three month physical exercise therapy. Neuroimage Clin. 9, 134–139. 10.1016/j.nicl.2015.08.004 26413476 PMC4556735

[B42] Fox M. D. Greicius M. (2010). Clinical applications of resting state functional connectivity. Front. Syst. Neurosci. 4:19. 10.3389/fnsys.2010.00019 20592951 PMC2893721

[B43] Francis G. (2012). Publication bias and the failure of replication in experimental psychology. Psychonom. Bull. Rev. 19, 975–991. 10.3758/s13423-012-0322-y 23055145

[B44] Gabis L. Shklar B. Baruch Y. K. Raz R. Gabis E. Geva D. (2009). Pain reduction using transcranial electrostimulation: A double blind active placebo controlled trial. J. Rehabil. Med. 41, 256–261. 10.2340/16501977-0315 19247545

[B45] Gabis L. Shklar B. Geva D. (2003). Immediate influence of transcranial electrostimulation on pain and β-Endorphin blood levels: an active placebo-controlled study. Am. J. Phys. Med. Rehabil. 82, 81–85. 10.1097/00002060-200302000-00001 12544752

[B46] Gagnon S. A. Wagner A. D. (2016). Acute stress and episodic memory retrieval: neurobiological mechanisms and behavioral consequences. Ann. N. Y. Acad. Sci. 1369, 55–75. 10.1111/nyas.12996 26799371

[B47] Gense de Beaufort D. Sesay M. Stinus L. Thiebaut R. Auriacombe M. Dousset V. (2012). Cerebral blood flow modulation by transcutaneous cranial electrical stimulation with Limoge's current. J. Neuroradiol. 39, 167–175. 10.1016/j.neurad.2011.06.001 21835468

[B48] Gilula M. F. (2007). Cranial electrotherapy stimulation and fibromyalgia. Expert Rev Med Devices 4, 489–495. 10.1586/17434440.4.4.489 17605684

[B49] Greicius M. D. Srivastava G. Reiss A. L. Menon V. (2004). Default-mode network activity distinguishes Alzheimer's disease from healthy aging: evidence from functional MRI. Proc. Natl. Acad. Sci. U.S.A. 101, 4637–4642. 10.1073/pnas.0308627101 15070770 PMC384799

[B50] Grupe D. W. Nitschke J. B. (2013). Uncertainty and anticipation in anxiety: an integrated neurobiological and psychological perspective. Nat. Rev. Neurosci. 14, 488–501. 10.1038/nrn3524 23783199 PMC4276319

[B51] Guleyupoglu B. Schestatsky P. Edwards D. Fregni F. Bikson M. (2013). Classification of methods in transcranial electrical stimulation (tES) and evolving strategy from historical approaches to contemporary innovations. J. Neurosci. Methods 219, 297–311. 10.1016/j.jneumeth.2013.07.016 23954780 PMC3833074

[B52] Gunther M. Phillips K. D. (2010). Cranial electrotherapy stimulation for the treatment of depression. J. Psychosoc. Nurs. Ment. Health Serv. 48, 37–42. 10.3928/02793695-20100701-01 20669869

[B53] Hamilton J. P. Furman D. J. Chang C. Thomason M. E. Dennis E. Gotlib I. H. (2011). Default-mode and task-positive network activity in major depressive disorder: implications for adaptive and maladaptive rumination. Biol. Psychiatry 70, 327–333. 10.1016/j.biopsych.2011.02.003 21459364 PMC3144981

[B54] Hampson M. Driesen N. R. Skudlarski P. Gore J. C. Constable R. T. (2006). Brain connectivity related to working memory performance. J. Neurosci. 26, 13338–13343. 10.1523/JNEUROSCI.3408-06.2006 17182784 PMC2677699

[B55] Harmony T. Fernández T. Silva J. Bernal J. Díaz-Comas L. Reyes A.. (1996). EEG delta activity: an indicator of attention to internal processing during performance of mental tasks. Int. J. Psychophysiol. 24, 161–171. 10.1016/S0167-8760(96)00053-0 8978441

[B56] Hermans E. J. Henckens M. J. A. G. Joëls M. Fernández G. (2014). Dynamic adaptation of large-scale brain networks in response to acute stressors. Trends Neurosci. 37, 304–314. 10.1016/j.tins.2014.03.006 24766931

[B57] Higgins J. P. T. Altman D. G. Gotzsche P. C. Juni P. Moher D. Oxman A. D.. (2011). The cochrane collaboration's tool for assessing risk of bias in randomised trials. BMJ 343:d5928. 10.1136/bmj.d5928 22008217 PMC3196245

[B58] Horvath J. C. Carter O. Forte J. D. (2014). Transcranial direct current stimulation: five important issues we aren't discussing (but probably should be). Front. Syst. Neurosci. 8:2. 10.3389/fnsys.2014.00002 24478640 PMC3901383

[B59] Howland R. H. (2014). Vagus nerve stimulation. Current Behav. Neurosci. Rep. 1, 64–73. 10.1007/s40473-014-0010-5 PMC4017164 24834378

[B60] Isotani T. Tanaka H. Lehmann D. Pascual-Marqui R. D. Kochi K. Saito N.. (2001). Source localization of EEG activity during hypnotically induced anxiety and relaxation. Int. J. Psychophysiol. 41, 143–153. 10.1016/S0167-8760(00)00197-5 11325459

[B61] Jacobs G. D. Benson H. Friedman R. (1996). Topographic EEG mapping of the relaxation response. Biofeedb. Self Regul. 21, 121–129. 10.1007/BF02284691 8805962

[B62] Kavirajan H. C. Lueck K. Chuang K. (2014). Alternating current cranial electrotherapy stimulation (CES) for depression. Cochrane Database Syst. Rev. 8:CD010521. 10.1002/14651858.CD010521.pub2 25000907 PMC10554095

[B63] Kim E. J. Pellman B. Kim J. J. (2015). Stress effects on the hippocampus: a critical review. Learn. Mem. 22, 411–416. 10.1101/lm.037291.114 26286651 PMC4561403

[B64] King A. P. Block S. R. Sripada R. K. Rauch S. Giardino N. Favorite T.. (2016). Altered default mode network (dmn) resting state functional connectivity following a mindfulness-based exposure therapy for posttraumatic stress disorder (ptsd) in combat veterans of Afghanistan and Iraq. Depress. Anxiety 33, 289–299. 10.1002/da.22481 27038410

[B65] Kirsch D. L. Chan S. C. (2013). Microcurrent and Cranial Electrotherapy Stimulator for Control of Anxiety, Insomnia, Depression and Pain (United States Patent and Trademark Office Patent No. US8612008B2).

[B66] Kirsch D. L. Gilula M. F. (2007). CES in the treatment of depression, part 2. Pract. Pain Manag. 6, 32–40.

[B67] Kirsch D. L. Nichols F. (2013). Cranial electrotherapy stimulation for treatment of anxiety, depression, and insomnia. Psychiatr. Clin. North Am. 36, 169–176. 10.1016/j.psc.2013.01.006 23538086

[B68] Kirsch D. L. Price L. R. Nichols F. Marksberry J. A. Platoni K. T. (2014). Military service member and veteran self reports of efficacy of cranial electrotherapy stimulation for anxiety, posttraumatic stress disorder, insomnia, and depression. US. Army Med. Dep. J. 2014:46–54. 25830798

[B69] Kirsch D. L. Smith R. B. (2000). The use of cranial electrotherapy stimulation in the management of chronic pain: a review. NeuroRehabilitation 14, 85–94. 10.3233/NRE-2000-14204 11455071

[B70] Klimesch W. (1999). EEG alpha and theta oscillations reflect cognitive and memory performance: a review and analysis. Brain Res. Rev. 29, 169–195. 10.1016/S0165-0173(98)00056-3 10209231

[B71] Knyazev G. G. (2012). EEG delta oscillations as a correlate of basic homeostatic and motivational processes. Neurosci. Biobehav. Rev. 36, 677–695. 10.1016/j.neubiorev.2011.10.002 22020231

[B72] Koenigs M. Ukueberuwa D. Campion P. Grafman J. Wassermann E. (2009). Bilateral frontal transcranial direct current stimulation: failure to replicate classic findings in healthy subjects. Clin. Neurophysiol. 120, 80–84. 10.1016/j.clinph.2008.10.010 19027357 PMC2650218

[B73] Koleoso O. N. Osinowo H. O. Akhigbe K. O. (2013). The role of relaxation therapy and cranial electrotherapy stimulation in the management of dental anxiety in Nigeria. IOSR J. Dent. Med. Sci. 10, 51–57. 10.9790/0853-1045157

[B74] Krupitsky E. M. Burakov A. M. Karandashova G. F. Katsnelson Ja S. Lebedev V. P. Grinenko A. Ja.. (1991). The administration of transcranial electric treatment for affective disturbances therapy in alcoholic patients. Drug Alcohol Depend. 27, 1–6. 10.1016/0376-8716(91)90080-I 2029855

[B75] Lande R. G. Gragnani C. (2013). Efficacy of cranial electric stimulation for the treatment of insomnia: a randomized pilot study. Complement. Ther. Med. 21, 8–13. 10.1016/j.ctim.2012.11.007 23374200

[B76] Lee J. Lee H. Park W. (2019). Effects of cranial electrotherapy stimulation on electrocephalogram. J. Int. Acad. Phys. Ther. Res. 10, 1687–1694. 10.20540/JIAPTR.2019.10.1.168732355172

[B77] Lee S. H. Kim W. Y. Lee C. H. Min T. J. Lee Y. S. Kim J. H.. (2013). Effects of cranial electrotherapy stimulation on preoperative anxiety, pain and endocrine response. J. Int. Med. Res. 41, 1788–1795. 10.1177/0300060513500749 24265330

[B78] Liss S. Liss B. (1996). Physiological and therapeutic effects of high frequency electrical pulses. Integr. Physiol. Behav. Sci. 31, 88–95. 10.1007/BF02699781 8809593

[B79] Lo B. Field M. J. (2009). Conflict of Interest in Medical Research, Education, and Practice. Washington, DC: National Academies Press. 20662118

[B80] Luethi M. Meier B. Sandi C. (2008). Stress effects on working memory, explicit memory, and implicit memory for neutral and emotional stimuli in healthy men. Front. Behav. Neurosci. 2:5. 10.3389/neuro.08.005.2008 19169362 PMC2628592

[B81] Martin G. N. Clarke R. M. (2017). Are psychology journals anti-replication? A snapshot of editorial practices. Front. Psychol. 8:523. 10.3389/fpsyg.2017.00523 28443044 PMC5387793

[B82] Mason J. W. (1968). A review of psychoendocrine research on the pituitary-adrenal cortical system. Psychosom. Med. 30, 576–607. 10.1097/00006842-196809000-00020 4303377

[B83] Maxwell S. E. Lau M. Y. Howard G. S. (2015). Is psychology suffering from a replication crisis? What does failure to replicate really mean? Am. Psychol. 70, 487–498. 10.1037/a0039400 26348332

[B84] McClure D. Greenman S. C. Koppolu S. S. Varvara M. Yaseen Z. S. Galynker I. I. (2015). A pilot study of safety and efficacy of cranial electrotherapy stimulation in treatment of bipolar II depression. J. Nerv. Ment. Dis. 203, 827–835. 10.1097/NMD.0000000000000378 26414234 PMC4892785

[B85] Mischoulon D. De Jong M. F. Vitolo O. V. Cusin C. Dording C. M. Yeung A. S.. (2015). Efficacy and safety of a form of cranial electrical stimulation (CES) as an add-on intervention for treatment-resistant major depressive disorder: a three week double blind pilot study. J. Psychiatr. Res. 70, 98–105. 10.1016/j.jpsychires.2015.08.016 26424428

[B86] Morriss R. Price L. (2020). Differential effects of cranial electrotherapy stimulation on changes in anxiety and depression symptoms over time in patients with generalized anxiety disorder. J. Affect. Disord. 277, 785–788. 10.1016/j.jad.2020.09.006 33065818

[B87] Morriss R. Xydopoulos G. Craven M. Price L. Fordham R. (2019). Clinical effectiveness and cost minimisation model of Alpha-Stim cranial electrotherapy stimulation in treatment seeking patients with moderate to severe generalised anxiety disorder. J. Affect. Disord. 253, 426–437. 10.1016/j.jad.2019.04.020 31103808

[B88] Moussa M. N. Steen M. R. Laurienti P. J. Hayasaka S. (2012). Consistency of Network Modules in Resting-State fMRI Connectome Data. PLoS ONE 7:e44428. 10.1371/journal.pone.0044428 22952978 PMC3432126

[B89] National Research Council (1974). An Evaluation of Electroanesthesia and Electrosleep: Report of the Ad Hoc Committee on Electrical Stimulation of the Brain. Report PB-241 305. Springfield, VA: National Technical Information Service.

[B90] Niedermeyer E. (1997). Alpha rhythms as physiological and abnormal phenomena. Int. J. Psychophysiol. 26, 31–49. 10.1016/S0167-8760(97)00754-X 9202993

[B91] Niedermeyer E. da Silva F. H. L. (2005). Electroencephalography: Basic Principles, Clinical Applications, and Related Fields. Philadelphia, PA: Lippincott Williams & Wilkins.

[B92] O'Connell N. E. Wand B. M. Marston L. Spencer S. Desouza L. H. (2011). Non-invasive brain stimulation techniques for chronic pain. A report of a Cochrane systematic review and meta-analysis. Eur. J. Phys. Rehabil. Med. 47, 309–326. 21494222

[B93] Oken B. S. Salinsky M. (1992). Alertness and attention: basic science and electrophysiologic correlates. J. Clin. Neurophysiol. 9, 480–494. 10.1097/00004691-199210000-00003 1361195

[B94] Peña C. Bowsher K. Costello A. De Luca R. Doll S. Li K.. (2007). An overview of FDA medical device regulation as it relates to deep brain stimulation devices. IEEE Trans. Neural Syst. Rehabil. Eng. 15, 421–424. 10.1109/TNSRE.2007.903973 17894274

[B95] Pert A. Dionne R. Ng L. Bragin E. Moody T. W. Pert C. B. (1981). Alterations in rat central nervous system endorphins following transauricular electroacupuncture. Brain Res. 224, 83–93. 10.1016/0006-8993(81)91118-5 6269708

[B96] Pessoa L. (2009). How do emotion and motivation direct executive control? Trends Cogn. Sci. 13, 160–166. 10.1016/j.tics.2009.01.006 19285913 PMC2773442

[B97] Pozos R. S. Richardson A. W. Kaplan H. M. (1971). Electroanesthesia: a proposed physiologic mechanism, in Neuroelectric Research, eds D. V. Reynolds and A. Sjodberg (Springfield, IL: Charles Thomas), 110–113.

[B98] Qiao J. Weng S. Wang P. Long J. Wang Z. (2015). Normalization of intrinsic neural circuits governing tourette's syndrome using cranial electrotherapy stimulation. IEEE Trans. Biomed. Eng. 62, 1272–1280. 10.1109/TBME.2014.2385151 25546850

[B99] Raichle M. E. (1998). Behind the scenes of functional brain imaging: a historical and physiological perspective. Proc. Natl. Acad. Sci. U.S.A. 95, 765–772. 10.1073/pnas.95.3.765 9448239 PMC33796

[B100] Robinovitch L. G. (1914). Electrical analgesia, sleep, and resuscitation, in Anesthesia, ed J. T. Gwathmey (New York, NY: Appleton).

[B101] Roh H. T. So W. Y. (2017). Cranial electrotherapy stimulation affects mood state but not levels of peripheral neurotrophic factors or hypothalamic- pituitary-adrenal axis regulation. Technol. Health Care 25, 403–412. 10.3233/THC-161275 27886020

[B102] Salar G. Job I. Mingrino S. Bosio A. Trabucchi M. (1981). Effect of transcutaneous electrotherapy on CSF β-endorphin content in patients without pain problems. Pain 10, 169–172. 10.1016/0304-3959(81)90192-5 6267542

[B103] Schacter D. L. (1977). EEG theta waves and psychological phenomena: a review and analysis. Biol. Psychol. 5, 47–82. 10.1016/0301-0511(77)90028-X 193587

[B104] Schooler J. (2011). Unpublished results hide the decline effect. Nature 470, 437–437. 10.1038/470437a 21350443

[B105] Schroeder M. J. Barr R. E. (2001). Quantitative analysis of the electroencephalogram during cranial electrotherapy stimulation. Clin. Neurophysiol. 112, 2075–2083. 10.1016/S1388-2457(01)00657-5 11682346

[B106] Shekelle P. G. Cook I. A. Miake-Lye I. M. Booth M. S. Beroes J. M. Mak S. (2018a). Benefits and harms of cranial electrical stimulation for chronic painful conditions, depression, anxiety, and insomnia. Ann. Intern. Med. 168, 414–421. 10.7326/M17-1970 29435567

[B107] Shekelle P. G. Cook I. A. Miake-Lye I. M. Mak S. Booth M. S. Shanman R. (2018b). The Effectiveness and Risks of Cranial Electrical Stimulation for the Treatment of Pain, Depression, Anxiety, PTSD, and Insomnia: A Systematic Review (VA ESP Project #05-226). Washington, DC: Department of Veterans Affairs. 29630193

[B108] Smith R. B. (2007). Cranial Electrotherapy Stimulation: Its First Fifty Years, Plus Three: A Monograph. Mustang, OK: Tate Publishing & Enterprises, LLC.

[B109] Smith R. B. (2013). CES Ultra: All Likert Scales [Retail]. CES ULTRA. Available online at: https://www.cesultra.com/blog/all-likert-scales/

[B110] Solomon S. Elkind A. Freitag F. Gallagher R. M. Moore K. Swerdlow B.. (1989). Safety and effectiveness of cranial electrotherapy in the treatment of tension headache. Headache 29, 445–450. 10.1111/j.1526-4610.1989.hed2907445.x 2668227

[B111] Southworth S. (1999). A study of the effects of cranial electrical stimulation on attention and concentration. Integr. Physiol. Behav. Sci. 34, 43–53. 10.1007/BF02688709 10381164

[B112] Sterne J. A. C. Savović J. Page M. J. Elbers R. G. Blencowe N. S. Boutron I.. (2019). RoB 2: a revised tool for assessing risk of bias in randomised trials. BMJ 366:l4898. 10.1136/bmj.l4898 31462531

[B113] Tatum W. O. (2014). Handbook of EEG Interpretation. New York, NY: Demos Medical Publishing. 10.1891/9781617051807

[B114] Toriyama M. (1975). Ear acupuncture anesthesia. Ear Throat 47, 497–501.

[B115] Vannorsdall T. D. van Steenburgh J. J. Schretlen D. J. Jayatillake R. Skolasky R. L. Gordon B. (2016). Reproducibility of tDCS results in a randomized trial: failure to replicate findings of tDCS-induced enhancement of verbal fluency. Cogn. Behav. Neurol. 29, 11–17. 10.1097/WNN.0000000000000086 27008245

[B116] Vedhara K. Hyde J. Gilchrist I. D. Tytherleigh M. Plummer S. (2000). Acute stress, memory, attention and cortisol. Psychoneuroendocrinology 25, 535–549. 10.1016/S0306-4530(00)00008-1 10840167

[B117] Vine S. J. Moore L. J. Wilson M. R. (2016). An integrative framework of stress, attention, and visuomotor performance. Front. Psychol. 7:1671. 10.3389/fpsyg.2016.01671 27847484 PMC5088191

[B118] Wagenseil B. Garcia C. Suvorov A. V. Fietze I. Penzel T. (2018). The effect of cranial electrotherapy stimulation on sleep in healthy women. Physiol. Meas. 39:114007. 10.1088/1361-6579/aaeafa 30475746

[B119] Weiss M. F. (1973). The treatment of insomnia through the use of electrosleep: an EEG study. J. Nerv. Ment. Dis. 157, 108–120. 10.1097/00005053-197308000-00003 4146811

[B120] Winick R. L. (1999). Cranial electrotherapy stimulation (CES): a safe and effective low cost means of anxiety control in a dental practice. Gen. Dent. 47, 50–55. 10321152

[B121] Wu W. J. Wang Y. Cai M. Chen Y. H. Zhou C. H. Wang H. N.. (2020). A double-blind, randomized, sham-controlled study of cranial electrotherapy stimulation as an add-on treatment for tic disorders in children and adolescents. Asian J. Psychiatr. 51, 101992. 10.1016/j.ajp.2020.101992 32145674

[B122] Yennurajalingam S. Kang D. H. Hwu W. J. Padhye N. S. Masino C. Dibaj S. S.. (2018). Cranial electrotherapy stimulation for the management of depression, anxiety, sleep disturbance, and pain in patients with advanced cancer: a preliminary study. J. Pain Sympt. Manag. 55, 198–206. 10.1016/j.jpainsymman.2017.08.027 28870799

[B123] Zaghi S. Acar M. Hultgren B. Boggio P. S. Fregni F. (2010). Noninvasive brain stimulation with low-intensity electrical currents: putative mechanisms of action for direct and alternating current stimulation. Neuroscientist 16, 285–307. 10.1177/1073858409336227 20040569

[B124] Zink B. J. (2001). Traumatic brain injury outcome: concepts for emergency care. Ann. Emerg. Med. 37, 318–332. 10.1067/mem.2001.113505 11223769

